# ASO Author Reflections: Walking on Thin Statistical Ice: Why Prehabilitation Evidence in Cancer Surgery May Be More Fragile Than We Think

**DOI:** 10.1245/s10434-025-18517-w

**Published:** 2025-10-09

**Authors:** Sarah Cook, Bora Kim, Xiaoqiu Liu, Mark Hancock, Michael Solomon, Cherry Koh, Sascha Karunaratne, Kate Alexander, Daniel Steffens

**Affiliations:** 1 Surgical Outcomes Research Centre (SOuRCe), Royal Prince Alfred Hospital, Sydney, NSW Australia; 2 Faculty of Medicine and Health, Central Clinical School, The University of Sydney, Sydney, Australia; 3 The Daffodil Centre, The University of Sydney, and Cancer Council NSW, Sydney, Australia; 4 Statistics Division, The George Institute for Global Health, University of New South Wales, Sydney, New South Wales Australia; 5 Faculty of Medicine, Health and Human Sciences, Macquarie University, Sydney, Australia; 6 Institute of Academic Surgery (IAS), Royal Prince Alfred Hospital, Sydney, Australia; 7 Career Development Fellow, Cancer Institute New South Wales, Sydney, Australia

## Past

Surgical oncology plays a central role in cancer care, with an estimated 12 million people globally requiring surgical treatment for cancer in 2015 alone.^1^ Despite its survival benefits, postoperative morbidity remains high, and studies have found that reducing postoperative complications can lead to improved long-term survival outcomes.^2^ This recognition has led to growing interest in prehabilitation, interventions designed to enhance postsurgical recovery by mitigating surgical stress through nutritional, functional, and psychological interventions.^3,4^ Although emerging evidence from randomized controlled trials (RCTs) appears promising, the robustness of this evidence has come under scrutiny, prompting the use of metrics such as the fragility index (FI), the reverse fragility index (RFI), and the fragility quotient (FQ) to assess the reliability of RCT findings.

## Present

A recent systematic review^5^ analyzed 76 RCTs evaluating the impact of prehabilitation on postoperative outcomes for patients undergoing thoracic, abdominal, and pelvic cancer surgeries. Across these trials, 544 postoperative complication outcomes were reported, yet only 25 were statistically significant. More concerning, the findings showed that the evidence supporting prehabilitation is fragile. In most cases, changing the outcome of just one patient could negate a statistically significant result (FI of 1), and changing the outcomes of four patients could render a non-significant result statistically significant (RFI of 4). Additionally, 64 % of the studies were assessed as having a high risk of bias, further undermining the reliability of the evidence base.

## Future

Robust scientific evidence should withstand small variations in outcomes to ensure confidence in clinical decision-making. Unfortunately, the current body of RCTs investigating prehabilitation in cancer surgery lacks this robustness. Given that minimal changes in patient outcomes can alter statistical significance, clinicians should be cautious about relying solely on these trials to guide practice. To address these limitations, future research should prioritize methodologic rigor and larger sample sizes. Additionally, researchers should incorporate comprehensive outcome assessment approaches, including clinical significance and minimal clinically important differences in patient-reported outcomes, to move beyond a narrow focus on statistical significance and medical parameters such as postoperative complications. Finally, whereas this review focused on prehabilitation, fragility analysis should be extended to Enhanced Recovery After Surgery (ERAS) trials, given their increasing clinical implementation. Such broader analysis could help determine whether fragile results reflect a broader methodologic issue in surgical RCTs or are specific to prehabilitation interventions.
